# Predictive power of PEN-3 cultural model in cervical cancer screening among women: a cross- sectional study in South of Iran

**DOI:** 10.1186/s12885-023-11240-3

**Published:** 2023-08-08

**Authors:** Sara Dadipoor, Azin Alavi, Zainab Kader, Shokrollah Mohseni, Hadi Eshaghi Sani Kakhaki, Nahid Shahabi

**Affiliations:** 1 Mother and Child Welfare Research Center, Hormozgan University of Medical Sciences, Bandar Abbas, Iran; 2 The Centre for Interdisciplinary Studies of Children, Families and Society, Faculty of Community and Health Sciences, University of the Western Cape, Bellville, South Africa; 3 Social Determinants in Health Promotion Research Center, Hormozgan Health Institute, Hormozgan University of Medical Sciences, Bandar Abbas, Iran

**Keywords:** Cervical cancer screening, Pap smear, Women, PEN-3 model, Iran

## Abstract

**Background:**

Cervical cancer (CC) can be prevented through early detection facilitated by screening as well as an early diagnosis and effective treatment of the precancerous lesions. The present research aimed to determine the predictors of cervical cancer screening (CCS) based on the PEN-3 model constructs.

**Methods:**

A cross-sectional study was conducted between September 2021- March 2022 with 840 women aged 15–49 in the city of Bandar Abbas, in the south of Iran, using a cluster sampling. The participants completed a valid and reliable self-administered questionnaire in person. The questionnaire included demographic characteristics, knowledge toward CC and the constructs of the PEN-3 model toward CCS. A multivariable logistic regression was used to determine the relationship and predictive power of model constructs with behavior as an outcome variable. The data were statistically analyzed in STATA_14.2_. The *p*-value < 0.05 was considered statistically significant.

**Results:**

A total of 810 questionnaires were analyzed (with a return of 95.63%). The mean and standard deviation of the participants’ age was 30.97 ± 5.80 years. Pearson correlation coefficient analysis of all constructs and CCS behavior was statistically significant (*P*-value < 0.05). The multivariable logistic regression analytic results were enablers toward CCS (coefficient: 0.275) and Nurturers toward CCS (coefficient: 0.182), perceptions toward CCS (coefficient: 0.077) and knowledge toward CC (coefficient: 0.048, marginal significant) were predictors of CCS behavior. For the internal validity of the designed prediction model, a sample of 1000 was selected using the bootstrap sample replacement method which demonstrated the accuracy of the model PEN-3 is about 75% in predicting CCS behavior.

**Conclusions:**

The results of the present research showed that personal factors such as perceptions and interpersonal factors such as enablers and nurturers toward CCS can predict CCS behavior. Therefore, in order to increase the acceptance of CCS in women, a set of intrapersonal and interpersonal factors should be taken into account.

**Supplementary Information:**

The online version contains supplementary material available at 10.1186/s12885-023-11240-3.

## Background

On a global scale, cervical cancer (CC) remains to be one of the most prevalent types of cancer among women. It is the fourth most prevalent cancer after breast, colorectal, and lung [1]. Annually, 528 000 new cases of cervical cancer are diagnosed globally, among which 200 000 lead to death [2].The incidence rate of CC in Iran is ever growing [3]. In this country, the incidence rate is 4.5 per 100,000 people. Every year, among every 123 women, one is afflicted with CC, and of every 100,000 women, nine die due to this cancer type [4].

CC is preventable through a timely detection using screening, early diagnosis and efficient management of precancerous lesions [5]. Of note is that prevention through screening only happens if high screening coverage is realized. It means that a high rate of eligible women use the screening tests in practice [6]. A high CCS coverage is considered as the major measure to reduce the incidence and mortality rates [7]. CCS coverage among eligible women in low- to middle-income countries (LMICs) is 19% on average in comparison to 63% in high-income countries (HICs) [8]. Sufficient reductions in mortality and morbidity occurring in HICs are mainly because of the higher uptake of screening, due to women’s better access to healthcare sources and their better CCS awareness [5].

Although CCS is one of the most effective ways to prevent CC, participation in the CCS programs in Iran is not ideal [9]. Studies have reported that Iranian women’s engagement in CCS programs is not satisfactory [10, 11]. Therefore, the low CCS challenge should urgently be resolved. It seems that the first step in solving this challenge is to identify the predictors of CCS. Once these predictors have been identified, goal-oriented and effective interventions can be designed to increase CCS. Meanwhile, health education theories can provide a more accurate understanding and preview of health behavior determinants. Theories play a significant role in explaining, clarifying and understanding the factors affecting health behaviors [12].

Several studies linked sociocultural barriers related to genital taboos with the decreased rate of CCS [13–15]. Cultural beliefs are considered a determinant of understanding health risks and health-promoting behaviors in different populations [16]. In some cultural contexts, there is a social stigma about women's health [17, 18]. Therefore, the researchers of this study employed a theory that recognize the value of the cultural context. Previous studies show that various models had been used to predict CCS [19, 20] but none of the educational models considered culture in predicting the factors affecting CCS as a focus and basis. The health provider is considered at the core of the PEN-2 model. This model is used to predict health-related behaviors in different studies on cancer [21, 22].

local and international studies have examined the factors affecting CCS. However, it is noteworthy that they have examined these factors in relation to demographic information [23–26]. Research about the predictors of CCS is sparse. The existing literature has either used models from health education theories [27, 28] or other models [19, 20, 29] to extract the predictors of CCS. None of them have used the PEN-3 to explore the predictors of CCS. Some of the studies that focused on CCS have explored the concepts of the PEN-3 model using a qualitative approach [30, 31]. To the best of our knowledge, no research has been conducted in the city of Bandar Abbas, Iran using the PEN-3 model to determine the predictors of CCS.

The high incidence of CC, the resultant mortality, the knowledge, attitude and poor performance of Iranian women concerning CC screening on the one hand [4, 32] and failure to launch HPV vaccination and the high cost of access to the vaccine on the other [33] point to the significance of conducting the present study. Also, this research is important considering the significant disease burden of CC and the major role that effective preventive strategies can play to facilitate an accurate understanding of the CCS. This is critical to design interventions to increase women’s engagement in the screening programs. Women’s health and preventive behaviors stem from society, culture, beliefs and opinions hence it has an impact on the acceptance of CCS behavior. For this reason, the current research aimed to determine the predictors of CCS based on the PEN-3 model in Bandar Abbas city in Hormozgan province, Iran.

### The PEN-3 cultural model

**Fig. 1:**
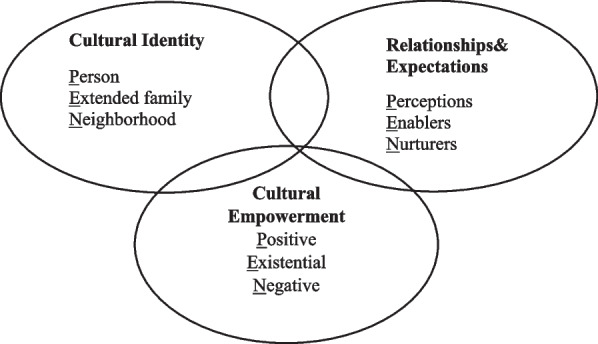
The theory of PEN-3 model

There are three primary domains in the PEN-3 cultural model: (1) Cultural Identity, (2) Relationships and Expectations, and (3) Cultural Empowerment. In each domain, three factors comprise the acronym PEN; Person, Extended Family, Neighborhood (Cultural Identity domain); Perceptions, Enablers, and Nurturers (relationship and expectation domain); Positive, Existential and Negative (Cultural Empowerment domain). The first domain emphasizes the intervention features of entry, which can realize at the level of persons (e.g., mothers or health care workers), extended family members (grandmothers), or neighborhoods (communities or villages). In the second domain, perceptions of or attitudes towards the health issues, the social or structural facilities including health care services that encourage or discourage efficient health-seeking practices, as along with the effect of family and kin in developing decisions on the successful management of health issues are examined. With the Cultural Empowerment domain, health issues are investigated initially through exploring positive beliefs and practices, investigating and emphasizing existential values and beliefs and those without any detrimental health effects, before exploring negative health practices that serve as obstacles. Thus, cultural beliefs and practices that affect health are explored along with solutions to health problems that can be further developed, those that do no harm are cherished, before finally facing practices that can be harmful and have adverse health effects [34] (Fig. 1). For the purpose of the present study, there is a focus on the PEN-3 cultural model constructs in the Relationships and Expectations domain and constructs in the Cultural Empowerment domain to explore CCS beliefs and values held by people who adopt instrumental roles in Iranian women’s lives and the enablers and nurturers that affect the development of CCS among them.

## Methods

### Study design and population

The present descriptive-analytical cross-sectional research was conducted in between September 2021- March 2022 in Bandar Abbas city, the capital city of Hormozgan province in the south of Iran.

The population included women aged 18–49 years old. The research location included comprehensive health service centers in Bandar Abbas city.

### Inclusion criteria

The participants had to be (a) 18 years of age or older, (b) have a minimum level of reading and writing literacy, (c) be sexually active but not pregnant, (d) have no CC and (e) provide consent to participate in the study. Incomplete questionnaires were excluded from the study.

### Sampling and sample size considerations

Via pmsampsize module in STATA_14.2_, the sample size was estimated. According to previous study, the prevalence of CCS behavior in Iran has been reported to be 50% [9]. In a study using the theory of planned behavior model, adjusted R 2 = 0.315 has been reported [35]. Predictive variables in this study included: knowledge, perceptions, enablers, nurturers. A minimum sample size of 385 was estimated. In this study, a cluster sampling method was used to modify the sample size design effect = 2/2. The final sample size was 847.

**Fig. 2:**
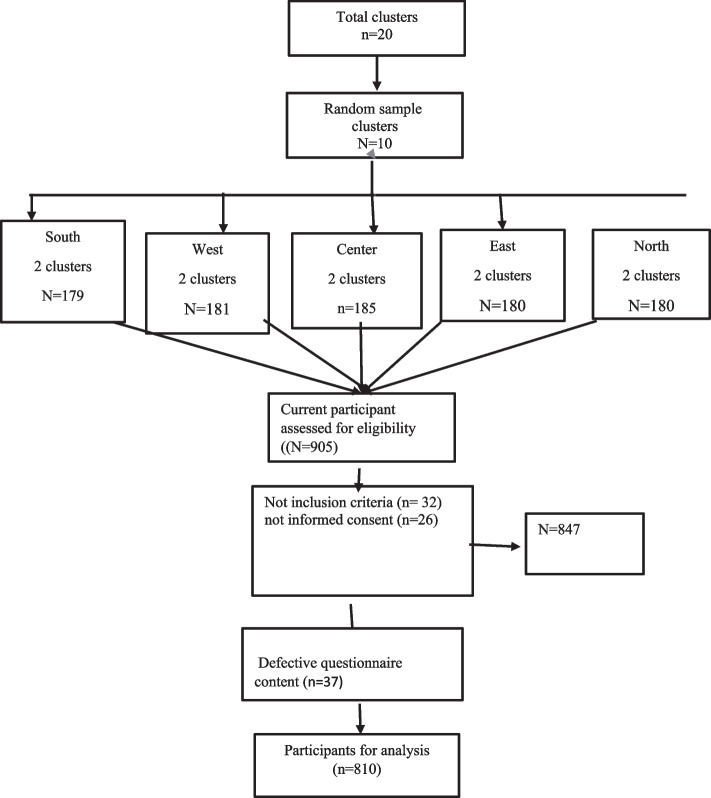
Flowchart for sample selection

For sampling, in the first step, 20 comprehensive health service centers of Bandar Abbas city were divided into 5 regions: North, South, East, West and Center. Four comprehensive health service centers were located in each geographical region. In the second step, the researchers randomly selected two centers from each region. In the third step, the first house from the third alley on the right side of the Comprehensive Health Service Center was selected as the head of the cluster, and the sampling continued until the cluster was complete. In each household, all eligible members were questioned. Finally, 905 households were visited, of which 847 received the questionnaires, and after removing incomplete questionnaires, 810 completed questionnaires were analyzed (Fig. 2).

### Measurements

The different sections of the self-administered questionnaire (Additional file 1) will be described in the following section along with the topics covered in each part:

#### Section 1

Demographic and reproductive information: age, marital status, education, occupation, socio-economic status (SES), Parity, history of sexually transmitted diseases, family history of cervical cancer.

Socio-economic status (SES) according to the distribution of the household crowding index (person/room ratio), and lowering crowdedness levels divided into upper, middle or lower SES (crowding index < 1, 1–2 and > 3 people per room) [36].

#### Section 2

This section assessed knowledge toward CC questions and PEN-3 constructs toward CCS [37] (Table 1).

**Table 1:** Description of the research instrument

Determinant	No. of Items (Format)	Scoring (Range)	Validity (Cronbach's alpha)	Item Example
Knowledge of CC	14 items/ 3 multiple choice	1. true 2. false 3. don't know	0.79	A history of CC in the family increases the risk of developing cancer in the rest of the family
Perceptions of CCS	16 items/5-point Likert Scale	Strongly Agree = 1, Agree = 2, No idea = 3, Disagree = 4, Strongly Disagree = 5	0.84	By doing a Pap smear test, any lesion and malignancy can be diagnosed and treated in time
Nurturers of CCS	11 items/5-point Likert Scale	1 = not at all, 2 = rarely, 3 = to a low degree, 4 = often, 5 = too often	0.85	To what extent does your husband encourage you to do a Pap smear test?
Enablers of CCS	12 items/5-point Likert Scale	1 = not at all, 2 = rarely, 3 = to a low degree, 4 = often, 5 = too often	0.78	To what extent do you have access to the health care unit to receive information and services related to the examination?

Each subscale was separately evaluated, and the total score was not estimated. Subscale scores were estimated for each participant. Higher scores represented more intense feelings about the particular construct.

### Data quality assurance

Seyrafi developed the questionnaire, it was given to health education experts and gynecologists to determine its content validity. Their feedback was used to modify the questionnaire.

To determine the reliability of the instrument, the test–retest method was used. To do this, the questionnaire was given to 30 people meeting the same conditions as the people under study in 10 days After that, each construct of the first test was compared with the second stage test. If the correlation coefficient between the first and second tests in each part was higher than 0.7, the questionnaire was confirmed. After that, to calculate the agreement between test and retest, the intraclass Correlation Coefficient (ICC (index was calculated. To calculate the agreement between the average test scores and the average retest scores, the ICC index value was estimated at 0.89 and the questionnaire was approved [37].

## Data collection

The data were collected using a self-administered questionnaire. The questionnaires were completed by the first author upon knocking the doors of participants’ houses and handing them the questionnaire. Each questionnaire took 15 minutes to complete. The participants completed the questionnaire at home at their convenience and then they returned them to the researcher at the same place. In most cases (more than 80 percent), only one woman from each household participated in the study and filled out the questionnaire., During this process, because the researcher was present at the site of data collection and double-checked the questionnaires to eliminate possible deficiencies (in case of not completing a number of questions, he gave the questionnaire to the participant again for completion). This is how missing data was addressed. However, in 37 cases, despite insistence on returning complete questionnaires, the participants failed to do so and, thus, incomplete questionnaires were excluded from the study. These questionnaires were then discarded. It is also noteworthy that the participants received no financial incentives.

### Ethical considerations

In order to collect data, the researcher visited the comprehensive health care centers with an official letter obtained from the research assistant of university and was, thus, introduced to the target setting of research. First, the researcher introduced himself to all participants and explained the objectives of study in a manner that was understandable to the participants. Then a written consent was obtained that included all the details about the research study. Participation in the study was voluntary and the anonymity and confidentiality of information was preserved. This study was approved by the Ethics Committee of Hormozgan University of Medical Sciences IR.HUMS.REC.1398.267.

### Outcome

Outcome: CCS behavior was determined by asking "Have you gone for a screening for cervical cancer in the past 3 years?"

Based on the response to this question, after collecting the data, we placed the people in two groups, including: 1- women who did the CCS in the past 3 years and 2- women who did not the CCS in the past 3 years.

Output: Scores obtained in the constructs of knowledge, perceptions, nurturers, enablers.

### Data management and analysis

For the quantitative variables knowledge, perceptions, enablers and nurturers, descriptive indicators (i.e., standard deviation, mean, percentage of mean) were used. For the qualitative variables age, marital status, education, occupation, economic status, parity, history of sexually transmitted diseases, family history of CC, CCS behavior, frequency (percentage) was used. Independent-samples *T*-test was used to compare the mean scores of the model constructs between two groups. The one group went for the screening test and the other one did not. Using Pearson correlation coefficient, the correlation matrix of model constructs was mapped. In order to relate the screening behavior with each construct, univariable a logistic regression analysis was used as well as multivariable logistic regression to determine the correlative and predictive power of the model constructs with behavior as the outcome variable. CCS during the past three years was coded 1 and no screening during the past 3 years was coded 2. The PEN-3 model, the current theoretical model, was comprised of three perceptions constructs, enablers and nurturers. Since, knowledge showed as a high significance in the univariate regression model, we added knowledge to these constructs to form the final model. To establish a prediction model, the two-state outcome variable was converted to probability and the linear equation of the predictor variables was obtained. Considering that we did not have another data source to test the model and its external validity, we had to test internal validity with a sample of 1000 with Bootstrap sample replacement method. Calibration chart and ROC chart were mapped. The data were statistically analyzed in STATA _14.2_. A *p*-value < 0.05 was considered statistically significant.

## Results

### Sample characteristics

Among the 847 eligible participants, 810 questionnaires were returned and subjected to the final analysis (response rate 95.63%). The mean and standard deviation of age was 30.97 ± 5.80. Most participants were 30–39 years old (52.2%), married (95.4%), of an average socio-economic status (74.1%), held a diploma (53.3%) and were housewives (91.13%). Most women were between 1–2 (76.3%) of parity, with a history of venereal disease (77.9%) and no family history of cervical cancer (96.8%). Of the total 262 (32.35%) women had done the CCS and 548 (67.65%) had not been screened for CS. Further demographic details are presented in Table 2.

**Table 2:** Sociodemographic and reproductive features of participants (*n* = 810)

Variable	Categories	Total N (%)	Screened for cervical cancer in the past 3 years N (%)	Not screened for cervical cancer in the past 3 years N (%)
Age	18–29	323 (39.9)	110 (42.0)	213 (38.9)
	30–39	423 (52.2)	141 (53.8)	282 (51.5)
	40–49	64 (7.9)	11 (4.2)	53 (9.7)
Marital status	Married	773 (95.4)	257 (98.1)	516 (94.2)
	Divorced/widowed	37 (4.6)	5 (1.9)	32 (5.8)
Educational level	Elementary/secondary school	148 (18.3)	16 (6.1)	132 (24.1)
	diploma	432 (53.3)	154 (58.8)	278 (50.7)
	University degree	230 (28.4)	92 (35.1)	138 (25.2)
Occupation	housewife	738 (91.1)	240 (91.6)	498 (90.9)
	Working	72 (8.9)	22 (8.4)	50 (9.1)
Socio-economic status	Upper	84 (10.4)	42 (16.0)	42 (7.7)
	Middle	600 (74.1)	210 (80.2)	390 (71.2)
	Lower	126 (15.6)	10 (3.8)	116 (21.2)
Parity	0	42 (5.2)	2 (0.8)	40 (7.3)
	1–2	618 (76.3)	239 (91.2)	379 (69.2)
	3–5	150 (18.5)	21 (8.0)	129 (23.5)
History of sexually transmitted disease	yes	631 (77.9)	227 (86.6)	404 (73.7)
	no	179 (22.1)	35 (13.4)	144 (26.3)
Family history of CC	yes	26 (3.2)	6 (2.3)	20 (3.6)
	no	784 (96.8)	256 (97.7)	528 (96.4)

The comparison of the mean score of the constructs in the two groups (screened and not screened) are shown in Table 3. This table shows that in all the constructs, the experimental group achieved higher scores. The highest percentage of the mean score that the participants achieved was related to the perceptions construct (*p*-value < 0.001).

**Table 3:** Mean ± standard deviation of PEN-3 constructs (*n* = 810)

Variable	Score range	Total		Screened for cervical cancer in the past 3 years		Not screened for cervical cancer in the past 3 years		p-value
		Mean ± SD	Average percentage	Mean ± SD	Average percentage	Mean ± SD	Average percentage	
Knowledge	0–14	8.42 ± 2.10	60.14	9.29 ± 1.64	66.35	8.01 ± 2.17	57.21	< 0.001
Perceptions	16–90	62.38 ± 3.95	69.31	63.69 ± 3.34	70.76	61.76 ± 4.07	68.62	< 0.001
Nurturers	11–55	33.01 ± 2.65	60.01	34.00 ± 2.63	61.81	32.54 ± 2.53	59.16	< 0.001
Enablers	12–60	37.26 ± 4.76	62.10	38.93 ± 4.12	64.88	36.47 ± 4.86	60.78	< 0.001

The correlation matrix of the constructs with each other is shown in Table 4. Based on the findings, there was a statistically significant relationship between all the constructs and CCS behavior (Table 4).

**Table 4:** Pearson’s correlation coefficient of the PEN-3 model constructs

Variables	Knowledge	Perceptions	Nurturers	Enablers
Knowledge	1			
Perceptions	.460 (< 0.001)	1		
Nurturers	.118 (.001)	.157 (< 0.001)	1	
Enablers	.393 (< 0.001)	.420 (< 0.001)	.110 (.002)	1

In Table 5, the relationship between PEN-3 model constructs and screening behavior was measured separately and all constructs showed to be significantly related to CCS.

**Table 5:** Univariate logistic regression analysis of PEN-3 constructs with cervical cancer screening

Variable	Coefficient	Std. Error	95.0% CI for coefficient		OR(95.0% CI)
			Lower Bound	Upper Bound	
Knowledge	.148	.024	.101	.194	1.16 (1.11–1.21)
Perceptions	.121	.018	.086	.157	1.13 (1.09–1.17)
Nurturers	.223	.032	.161	.285	1.25 (1.17–1.33)
Enablers	.37	.048	.277	.464	1.45 (1.32–1.59)

Table 6 shows the relationship between the PEN-3 model constructs in multivariate logistic regression and the screening behavior. As shown in Table 6, enablers, nurturers and perceptions predict CCS. Furthermore, knowledge was a marginally significant predictor. The fit indices of the model show that the model has a relatively good predictability.

**Table 6:** Multivariate logistic regression analysis of PEN-3 constructs with cervical cancer screening

Variable	Coefficient	Std. Error	95.0% CI for coefficient		OR (95.0% CI)
			Lower Bound	Upper Bound	
Knowledge	.048	.027	-.01	.1	1.05 (0.99–1.10)
Perceptions	.077	.02	.04	.12	1.08 (1.04–1.12)
Nurturers	.182	.033	.12	.25	1.20 (1.12–1.28)
Enablers	.275	.051	.17	.37	1.32 (1.19–1.45)
Constant	-15.11	1.82	-18.68	-11.54	2.73 (7.71–9.70)
Mean (SD) dependent variable				0.32 (0.47)	
Pseudo R-squared				0.138	
R^2^ Cox-Snell				0.159	
R^2^ Nagelkerke				0.222	
R^2^ McFadden				0.138	
R^2^ McFadden(adj)				0.128	
Akaike crit. (AIC)				889.286	
Bayesian crit. (BIC)				912.771	
**Calculation of linear predictor for PEN-3 model = (Knowledge × 0.048) + ( Perception × 0.077) + ( Nurturers × 0.182) + (Enablers × 0.275)**					

**Fig. 3:**
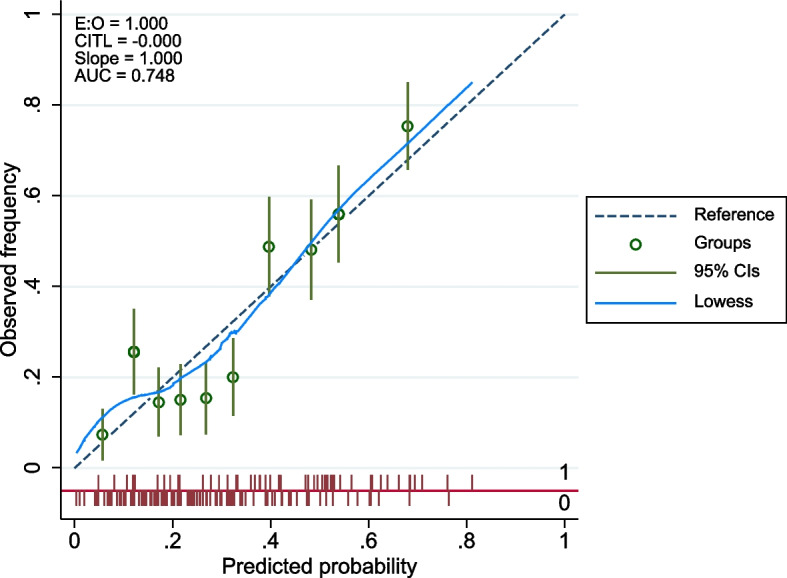
Internal calibration plot for CCS from the PEN-3 model

**Fig. 4:**
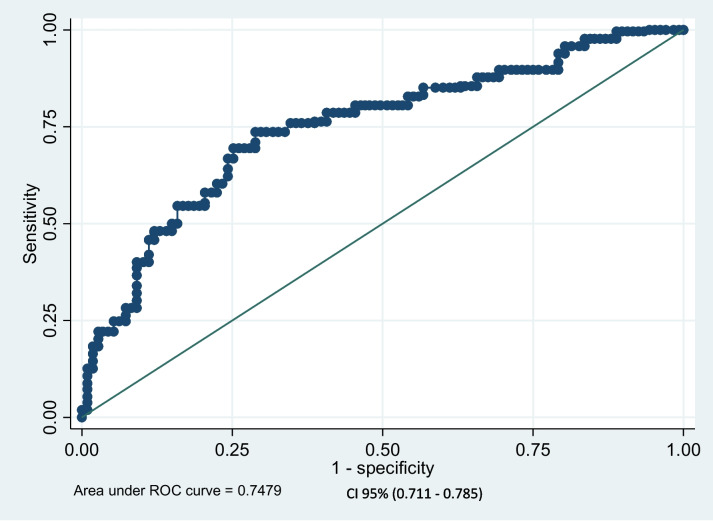
ROC curve plot for CCS from the PEN-3 model

For the internal validity of the prediction model, a sample of 1,000 was selected using the bootstrap sample replacement method, and then the calibration chart (Fig. 3) and the ROC curve (Fig. 4) were drawn. As the fit index of these two figures showed, the accuracy of the PEN 3 model was confirmed. This model is capable of predicting 75% of CCS behavior.

## Discussion

Cervical Cancer Screening behaviors are low in women, and factors such as perceptions, enablers, and nurturers can predict the cause.

Presumably, knowledge of the causal factors and prevention methods is essential to circumvent CC. Knowledge affects individuals’ decision-making. Knowing these factors can affect individuals to avoid or overcome them and, hence escape affliction with the disease.

The results showed that 60% of women achieved an average knowledge score of the signs and symptoms of CC, causes, risk factors, prevention and treatment alternatives. Researchers reported different levels of knowledge in different studies. A study in India reported that 82.9% of participants had heard of CC, 51% were aware that the disease was preventable, and 2.3% knew it could be detected at an early stage [38]. A study in Ethiopia reported that 57.8% of the participants had heard about CC, 23.4% were aware of the symptoms, prevention, early diagnosis and treatment of the disease [39]. The findings of another study showed that 46.3% of the participating women had a low level of knowledge about vulnerable groups, risk factors, signs and symptoms and prevention methods of cervical cancer [40]. This difference in results can be attributed to the features of research population, level of questionnaire content and different sample size in different studies.

In the present study, 262 (32.34%) of the participants were screened for CC. In some studies, the screening level of CC was lower than the present study [40, 41] and in some others, the screening level of CC was higher than the present study [27]. The different socio-demographic features of the participants can be one potential reason for this contradiction and the sample size could be one potential reason for this discrepancy, as the sample size in Akinlotan's study was 4276 women living in 47 cities. The lack of CCS in the present study women participants can be attributed to various factors. It seems that education and information about CCS is limited therefore women have less access to this information. It can be also argued that barriers such as financial costs, social and cultural barriers, lack of access to health services could have contributed to the low rate of screening among women.

The present findings showed a statistically significant relationship between knowledge and the adoption of CCS. This finding points to the fact that knowledge about the causes, risk factors, and preventive or therapeutic alternatives for CC has led to an increase in the acceptance of CCS. Chisale in his study stated that, a higher level of knowledge was associated with more CCS [5]. As the results of Isabirye and Karimy studies showed, the chances of screening for CC in women increased with increasing knowledge [28, 42]. Arguably, knowledge is one of the most important aspects of behavior information. It will be easier to adopt new behaviors if it is based on correct knowledge and awareness. It should be noted that knowledge was not a predictor of CCS behavior in multiple regression analysis. Knowledge is essential particularly for rational decision-making of any health behavior but it is not sufficient. A study showed that knowledge can predict the intention to perform CCS through influencing the attitude [43]. Therefore, in the current research, it seems that although knowledge did not predict CCS behavior, it probably indirectly led to an increase in CCS through influencing perceptions and other constructs of the PEN3 model.

Based on the present findings, enablers are the strongest predictors of CCS behavior. Enablers in different studies were effective and useful in increasing the acceptance of CCS: having enough time ،affordability [26, 44, 45], the reasonable cost of Pap smear tests, health workers’ information conveyed in the native language, distribution of printed materials in the native language, and the use of culturally sensitive media [5, 46]. This finding is indicative of the fact that enablers play a decisive role in encouraging women to undergo CCS. Some women may not go for the screening due to the perceived barriers and lack of enablers despite having sufficient knowledge and a positive perception. In another study, despite perceived behavioral control, women did not screen for CC due to the presence of uncontrollable barriers [47]. Also, it was reported in the United States that low education, lack of health insurance, unemployment, and lower income influenced the non-adherence to CCS [29]. The present researchers believe that these barriers are the same enablers that act as a catalyst to adopt a healthy behavior.

Our findings showed that nurturers were among the predictors of CCS behavior. Similarly, nurturers such as spouse, parents, health workers and friends play an important role in increasing women's knowledge and encouraging them to go for a CCS [20, 29, 48, 49]. We also found that women who stated that their family, friends and doctor expected them to do the CCS were more likely to perform CCS in practice. Therefore, it is important for interventionists to ensure that women receive copious support from their significant others. It is further suggested to design effective programs to increase the acceptance of CCS by identifying women’s support systems and intervene with them as well.

The results of this study showed that the effect of perceptions on CCS behavior is statistically significant. A positive perceptions towards CCS is significantly associated with women's willingness to undergo CCS [20, 46]. In some studies, a positive attitude towards CCS is an important predictor of women's willingness to undergo CCS [48, 50]. In contrast to our findings, In the Roncancio study, attitude was insignificant in predicting the intention to screen for CC [47]. To explain these divergences, we can say that certain cultural contexts are marked by social stigma about women's health [17, 18] particularly among Muslim women, for whom talking about reproductive health is considered a taboo and can be embarrassing [51]. In several countries, women's health is not attended carefully as it should, which discourages women from receiving preventive health care services such as CCS [18]. Difference in the context/circumstances under which the behavior is expected to be shown and the region/place where the study is conducted are among other reasons for this discrepancy in findings in the existing literature.

### Strengths and limitations

To the best of the authors' knowledge, this is the only study describing women's beliefs about CC and Pap smear using PEN-3 in the southern region of Iran, where CCS coverage is low. Our research identifies predictors that are the potential targets for interventions to improve screening acceptance among women in the region. One strength of this study is the use of the theoretical model framework. Another strength is the sufficient and suitable sample size. Although this study provides important information, it has certain limitations too. Since the present study is the first to examine the use of PEN3 in predicting CCS in women in southern Iran, it is not possible to make any comparison across studies, so more research should be done in future to confirm the appropriateness of the model. The low generalizability of the findings to women in other regions was another limitation of the current research. However, attempts were made to partly reduce the effect of this limitation with a sufficient sample size and selection of women with different demographic information. The present data were completed in the form of self-report. Thus, maybe women provided socially desirable answers. Attempts were made to lower the effects of this bias by highlighting the confidentiality of the information the participants provided. Also, due to the fact that no other database was available in this study, internal validation was used to check the validity of the model. This study had a cross-sectional design, which made it less suitable to draw definite conclusions about causal relationships. Therefore, it is suggested to rely on more longitudinal experimental studies in future. The questionnaire was completed by women who were willing to participate in the study, so the results of this study cannot reflect the opinions of people who refused to participate in this study. Another limitation of this study was that according to the purpose of study, which was to explore the predictability of the PEN3 model, we did not include demographic information in the predictive model and only used the model constructs. In future studies, the authors will pay more attention to this issue. Furthermore, based on one of our questions about screening behavior, after collecting the data, we assigned the participants to two groups, those who did and those who did not the CCS. Therefore, unfortunately, it is not possible to mention the response rate of the two groups.

## Conclusion

The knowledge obtained from the present research can help to develop a goal-oriented and effective intervention to increase the acceptance of CCS. The results of the present research show that personal factors such as knowledge, perceptions and interpersonal factors such as enablers and nurturers can be effective in increasing the screening rate of CC. In order to increase the acceptance of CCS in women, a set of intra-personal and interpersonal factors should be taken into account.

### Supplementary Information

**Additional file 1. **Questionaire.

## Data Availability

The datasets used and/or analysed during the current study are available from the corresponding author on reasonable request.

## Abbreviations

CCS: Cervical cancer screening
CC: Cervical Cancer
