# Supplementary material for: Predictive power of PEN-3 cultural model in cervical cancer screening among women: a cross- sectional study in South of Iran
Source: BMC Cancer. 2023 Aug 8;23:730. doi: 10.1186/s12885-023-11240-3 (PMC10408223; doi:10.1186/s12885-023-11240-3)
Supplement: Supplementary file 1 — Additional file 1. Questionaire. [file 12885_2023_11240_MOESM1_ESM.docx]

**Questionnaire**

***Knowledge***

1.An increase in age is accompanied by higher chances of affliction with cervical cancer.

Yes No Don’t know

2. Marriage at an early age (<18) increases the chances of affliction with cervical cancer.

Yes No Don’t know

3. The history of cervical cancer in family increases the chances of affliction with cervical cancer in other family members.

Yes No Don’t know

4. Doing the Pap test after marriage is recommended.

Yes No Don’t know

5. Cervical cancer is preventable.

Yes No Don’t know

6. The Pap test is a preterm diagnostic test for cervical cancer.

Yes No Don’t know

7. The best time to have the Pap test is two weeks after the menstrual period begins.

Yes No Don’t know

8. The Pap test is just essential for women with unnatural bleedings.

Yes No Don’t know

9. Taking contraceptives is associated with affliction with cervical cancer.

Yes No Don’t know

10. The lack of genital hygiene increases the chances of affliction with the cervical cancer.

Yes No Don’t know

11. Vitamin A and C deficiency in the diet increases the chances of affliction with cervical cancer.

Yes No Don’t know

12. Folic acid deficiency in diet increases the chances of affliction with cervical cancer.

Yes No Don’t know

13. Tobacco consumption (cigarettes, hookahs, ...) is associated with cervical cancer.

Yes No Don’t know

14. Having multiple sex partners increases the chances of affliction with cervical cancer.

Yes No Don’t know

***Perceptions***

1.The Pap test puts my mind at rest.

Strongly disagree Disagree No idea Agree Strongly agree

2. Seeing or hearing of people afflicted with the cervical cancer encourages e to go for the test.

Strongly disagree Disagree No idea Agree Strongly agree

3. I think doing the Pap test and regular visits to a gynecologist indicate women’s health.

Strongly disagree Disagree No idea Agree Strongly agree

4. The Pap test is capable of an early diagnosis of any malignancy.

Strongly disagree Disagree No idea Agree Strongly agree

5. The Pap test is effective in reducing the cases with cervical cancer and the mortality it accounts for.

Strongly disagree Disagree No idea Agree Strongly agree

6. Even if there are chances that I get afflicted with the cervical cancer, I will not go for the Pap test.^*^

Strongly disagree Disagree No idea Agree Strongly agree

7. I do not like to add to my concerns by having the Pap test. ^*^

Strongly disagree Disagree No idea Agree Strongly agree

8. I will never get afflicted with the cervical cancer so I think the Pap test is not urgent^*.^

Strongly disagree Disagree No idea Agree Strongly agree

9. I have fears of diagnosing tumors so I do not go for a Pap test.^*^

Strongly disagree Disagree No idea Agree Strongly agree

10. Having the Pap test in the presence of a physician is embarrassing to me^*^.

Strongly disagree Disagree No idea Agree Strongly agree

11. I think cervical cancer is not preventable. ^*^

Strongly disagree Disagree No idea Agree Strongly agree

12. I think the Pap test is useless^*^

Strongly disagree Disagree No idea Agree Strongly agree

13.I think affliction with cervical cancer is one’s fate and is not preventable^*^.

Strongly disagree Disagree No idea Agree Strongly agree

14. I think free of charge preterm diagnostic services for cervical cancer are not reliable^*^.

Strongly disagree Disagree No idea Agree Strongly agree

15. If I am afflicted with the cervical cancer, I prefer not to know about it^*^.

Strongly disagree Disagree No idea Agree Strongly agree

16. I think the Pap test is easier than treating the cervical cancer.

Strongly disagree Disagree No idea Agree Strongly agree

***Enablers***

1.To what extent do you have access to health care centers to receive the examination information and services?

To a great extent Much Not much Almost not Not at all

2. To what extent, are the staff in the health care centers of the doctor's office qualified to do the Pap test?

To a great extent Much Not much Almost not Not at all

3. Do you face any financial problem to visit a health care center and pay for the Pap test^*^?

To a great extent Much Not much Almost not Not at all

4. To what extent is having an insurance card effective in visiting a doctor and doing the Pap test?

To a great extent Much Not much Almost not Not at all

5. To what extent do the work load and time limit prevent you from doing the Pap test^*^?

To a great extent Much Not much Almost not Not at all

6. To what extent are your cultural values and beliefs effective in your decision to go for the Pap test^*^?

To a great extent Much Not much Almost not Not at all

7. To what extent do you care less about the Pap test because of neglect or absent-mindedness^*^?

To a great extent Much Not much Almost not Not at all

8. To what extent does the crowdedness of the healthcare centers and the staff’s behavior affect your decision about taking the Pap test^*^?

To a great extent Much Not much Almost not Not at all

9. To what extent does the high cost of the Pap test affect your decision to take the Pap test^*^?

To a great extent Much Not much Almost not Not at all

10. To what extent does the distance from the place of administering the Pap test (the healthcare center, hospital clinic or doctor’s office) affect your decision to take the Pap test^*^?

To a great extent Much Not much Almost not Not at all

11. To what extent does the distance from the lab where you hand in the sample affect your decision to take the Pap test^*^?

To a great extent Much Not much Almost not Not at all

12. To what extent does the embarrassment involved in the vaginal clinical examination by a doctor or midwife affect your decision to take the Pap test^*^?

To a great extent Much Not much Almost not Not at all

***Nurturers***

1.To what extent does your husband disapprove of the Pap test^*^?

To a great extent Much Not much Almost not Not at all

2. To what extent does your husband disapprove of your presence in a doctor’s office or healthcare center for the Pap test^*^?

To a great extent Much Not much Almost not Not at all

3. To what extent does your husband encourage you to do the Pap test?

To a great extent Much Not much Almost not Not at all

4. To what extent do your family members (e.g., mother, sister, etc.) approve of your having a Pap test?

To a great extent Much Not much Almost not Not at all

5. To what extent do your husband’s family members approve of your having a Pap test?

To a great extent Much Not much Almost not Not at all

6. To what extent do your friends encourage you to go for a Pap test?

To a great extent Much Not much Almost not Not at all

7. To what extent do the health staff encourage you to do the Pap test?

To a great extent Much Not much Almost not Not at all

8. To what extent did your visiting physician encourage you to do the Pap test?

To a great extent Much Not much Almost not Not at all

9. To what extent do the religious characters (the Imam Jema’ah, Imam Jumah) encourage you to do the Pap test?

To a great extent Much Not much Almost not Not at all

10. To what extent do the influential characters in family encourage you to do the Pap test?

To a great extent Much Not much Almost not Not at all

11. To what extent do the influential characters in family encourage you to do the Pap test?

To a great extent Much Not much Almost not Not at all

***Behavior***

1.Have you ever had the Pap test?

Yes No

****All marked questions are reverse marked****.*
